# Evaluation of occlusion and disocclusion time in immediately loaded implant overdentures retained by two splinted versus non-splinted interforaminal implants: A randomized clinical trial

**DOI:** 10.1186/s12903-026-08855-w

**Published:** 2026-06-22

**Authors:** Omnia M. Refai, Ahmed Mostafa Abdelfattah Mohamed, Amr Mohamed Ali El-Fiky

**Affiliations:** 1 Lecturer of Oral and Maxillofacial Prosthodontics, Faculty of Dentistry, Ain Shams University, Cairo, Egypt; 2 Oral and Maxillofacial Prosthodontics department, Faculty of Dentistry, Ain Shams University, Cairo, Egypt; 3 Removable Prosthodontics department, Faculty of Oral and Dental Medicine, Nahda University, Benisuef, Egypt; 4 Faculty of Dentistry, Galala University, Galala, Egypt

**Keywords:** Implant, Prosthetic, Immediate Dental Implant Loading, Splints

## Abstract

**Background:**

Mandibular complete dentures often lack stability, leading to prolonged occlusion and disocclusion times. Immediate-loading implant overdentures can improve functional control, but evidence comparing splinted and non-splinted two-implant overdentures is limited. This randomized clinical trial aimed to evaluate occlusion and disocclusion times in patients receiving immediately loaded mandibular overdentures retained by two implants, comparing ball attachments and intraoral welded bar attachments, with conventional complete dentures serving as the control group.

**Methods:**

Thirty-six completely edentulous patients were randomized into three equal groups (*n* = 12 each): conventional complete dentures (CD), two-implant overdentures retained with ball attachments (IB), and two-implant overdentures retained with an intraoral welded bar (IW). Occlusion (OT) and right and left disocclusion times (DR, DL) were measured using the T-Scan system on the day of implant placement (T1) and after three months (T2). Data were normally distributed; Mixed ANOVA was used to assess the effects of time, treatment, and their interaction. Paired t-tests were additionally used for within-group comparisons. Post hoc Tukey tests were performed for pairwise comparisons, with Bonferroni correction applied for multiple testing. Statistical significance was set at *P* < 0.05.

**Results:**

At T1, the CD group exhibited significantly longer occlusion time (OT: 0.52 ± 0.07 s), right disocclusion time (DR: 0.57 ± 0.04 s), and left disocclusion time (DL: 0.58 ± 0.04 s) compared with the IB (OT: 0.35 ± 0.06 s; DR: 0.40 ± 0.04 s; DL: 0.40 ± 0.02 s) and IW (OT: 0.29 ± 0.02 s; DR: 0.36 ± 0.03 s; DL: 0.42 ± 0.01 s) groups (*P* < 0.001), indicating that implant-supported overdentures achieved more efficient occlusal contacts. No significant differences were observed between IB and IW. At T2, the pattern persisted, with the CD group showing a significant reduction in OT, DR, and DL over time (*P* < 0.05), reflecting improved OT and DT of CD, whereas both implant overdenture groups maintained stable OT and DT.

**Conclusions:**

Within three months of follow-up, immediately loaded mandibular overdentures retained by two implants showed similar OT and DT for IW and IB, while both groups showed shorter times compared to CD under the tested conditions.

**Trial registration:**

This randomized clinical trial was registered prospectively on Clinical Trials.gov with the Identification number: NCT06376019 on 19/04/2024.

**Supplementary Information:**

The online version contains supplementary material available at 10.1186/s12903-026-08855-w.

## Introduction

Full-arch implant-supported prostheses are considered the optimal treatment for completely edentulous patients, offering superior function, stability, and patient satisfaction. However, their application may be limited by systemic health conditions, surgical contraindications, or financial constraints [1]. Consequently, conventional removable complete dentures remain a widely used option due to their low cost and ability to restore basic aesthetics and function [2]. Nevertheless, complete dentures, particularly mandibular ones, are often associated with challenges such as poor stability due to residual ridge resorption [3]. In response to these limitations, the McGill Consensus has established the two-implant mandibular overdenture, placed in the interforaminal region with the potential for immediate loading, as the standard of care [4, 5]. This approach provides a cost-effective solution that significantly enhances denture retention, stability, and overall function [5].

Implant-assisted overdentures can use various attachment systems, including unsplinted (Ball) and splinted (bar) designs [6]. Ball attachments are widely studied and well established for implant retention, valued for their strong retention, ease of use, and ability to accommodate minor discrepancies in implant angulation [7–9]. However, they can lose retention over time due to wear of internal components [9]. Bar and clip systems provide strong retention and effective distribution but are associated with higher cost and technique-sensitive fabrication requirements [10].

The timing of prosthetic loading significantly affects the success of overdentures. While Brånemark originally recommended delayed loading to ensure osseointegration [11, 12], advances now allow immediate loading, placing a provisional prosthesis within 48 h, with high success rates and faster functional restoration [12–15]. Immediate loading of two-implant retained overdentures has shown excellent survival outcomes when implant stability is sufficient and functional forces are controlled [16–18]. Among immediate loading techniques, intraoral welding introduced by Dr. Pierluigi Mondani in the 1970s has shown promise [19]. Intraoral welding provides rigid splinting of titanium implants, enhancing primary stability, minimizing micromotion, and improving load distribution, which benefits peri-implant tissues [19–22]. Although technique-sensitive and requiring specialized equipment, recent advances and adoption of immediate loading protocols have renewed interest in this approach for implant overdentures [21, 22].

The T-Scan system provides an objective digital method for evaluating occlusal function by measuring occlusion time (OT) and disocclusion time (DT) [23]. Shorter OT indicates faster and more simultaneous tooth contact, reflecting improved occlusal equilibration, while shorter DT reduces contractile muscle activity during mandibular excursions and may decrease stress on the temporomandibular joints [23, 24]. Changes in these parameters have been reported following implant prosthetic rehabilitation, indicating functional adaptation to implant-supported prostheses [24]. Evaluating OT and DT under immediate loading is clinically relevant, as early occlusal stability can influence implant loading patterns, masticatory efficiency, and patient comfort during the initial adaptation period [25].

Both splinted bar and unsplinted ball attachments demonstrate high success rates and stable bone levels over 1–4 years, with splinted designs offering better load distribution and unsplinted attachments being easier to maintain [14, 16, 17]. Despite extensive evaluation of biological and prosthetic outcomes, evidence comparing the functional occlusal performance of splinted and unsplinted immediate-loading overdentures remains scarce. [26–29]. To date, no study has objectively evaluated OT and DT using the T-Scan system in immediately loaded mandibular overdentures, comparing splinted (intraoral welded bar–retained) and unsplinted (ball-retained) designs. Therefore, this randomized clinical trial aimed to evaluate OT and DT in patients receiving immediately loaded mandibular overdentures retained by two implants, comparing ball attachments (IB) and intraoral welded bar attachments (IW), with conventional complete dentures (CD) serving as the control group. The primary outcome was the difference in OT and DT between the IB and IW groups, with secondary outcomes comparing them to the CD group. The null hypothesis stated that no significant differences exist in OT and DT among the IB, IW, and CD groups.

## Materials and methods

**Fig. 1:**
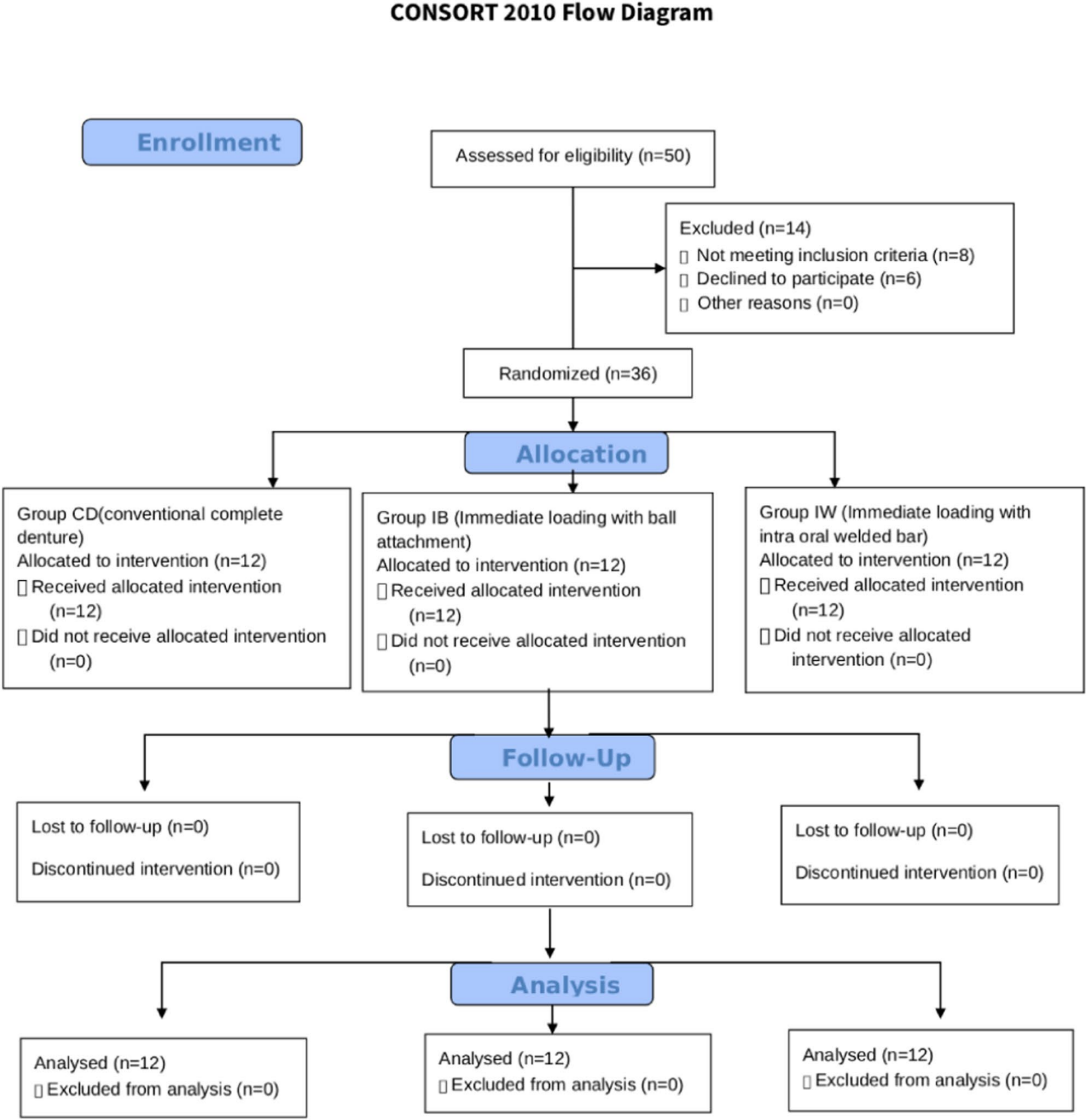
CONSORT 2010 flow diagram summarizing participant recruitment, group allocation, follow-up, and inclusion in the final analysis for the three study groups

The current study was designed as a parallel, randomized controlled clinical trial, following the CONSORT 2010 guidelines. (Fig. 1) The trial was registered prospectively on Clinical Trials.gov with the Identification number: on 19/04/2024, with a start date on 25/04/2024 and completed on 15/09/2024.

Thirty-six completely edentulous patients were selected from a governmental institution to share in the current study (twelve patients per group). Sample size calculation was based on a 95% confidence interval and a power 80% with an α error 5% (MedCalc^®^ version 12.3.0.0 program, Ostend, Belgium) in the light of a study conducted by Kabbua P et al. [27]. Using the mean difference (5.28%) and standard deviation (5.79%) for the values of maximum occlusal contact force before implant placement (84.14%) and follow-up period (89.77%), an effect size of 0.97 was yielded. Patients were randomized using a computer-generated list (Random Alloc, Software Informer, Informer Technologies Inc) and allocated into three groups: CD, IB, and IW. Participants were randomly assigned in a 1:1:1 ratio using opaque sealed envelopes, with allocation performed by a postgraduate student who was the only person aware of the codes. Blinding of participants and clinicians was not possible due to the visible differences in prosthetic design; only the operator evaluating the T-Scan recordings was blinded. All participants received conventional maxillary complete dentures, and the mandibular prostheses were fabricated as follows: the CD group received conventional mandibular complete dentures, while the IB and IW groups received immediately loaded mandibular overdentures retained by two interforaminal implants, using ball attachments and intraoral welded bars, respectively. The occlusal scheme was standardized for all participants using lingualized balanced occlusion.

Completely edentulous participants with eligibility criteria matching those registered in the clinical trial were enrolled. Those with a Class I maxillomandibular relationship, adequate inter-arch space, and sufficient bone width and height in the interforaminal region, who were non-smokers, were included in the study. Conversely, individuals with uncontrolled systemic diseases (e.g., diabetes mellitus), heavy smoking habits, temporomandibular joint disorders (TMD), need for extensive bone grafting, pregnancy, bisphosphonate therapy, and limited mouth opening were excluded. Fabrication of complete dentures was performed conventionally for all participants. Primary impressions were made by irreversible hydrocolloids (Cavex Cream Alginate, Cavex Holland BV, Haarlem, The Netherlands), followed by Zinc Oxide secondary impressions (Cavex Outline, Cavex Holland BV, Haarlem, The Netherlands). Facebow, protrusive, and centric relation records were mounted on a semi-adjustable articulator. Lingualized balanced occlusion was selected as the occlusal scheme. Dentures were processed using heat-cured prepolymerized acrylic resin (Acrostone Dental and Medical Supplies, Cairo, Egypt) and a conventional compression molding technique. Laboratory remounting then followed, and dentures were delivered to the participants [30].

For implant placement, the mandibular denture was duplicated into a clear acrylic surgical stent with radiopaque markers (Meta Biomed, Chungcheong Buk, South Korea) positioned at the canine regions. Cone Beam Computed Tomography (CBCT) (i-CAT™ FLX V8, Kavo, California, USA) were obtained for all participants while wearing the stent to evaluate bone quality and quantity. Bone density ranged from 800 to 1200 HU (D1–D2).

Implants were inserted in the mandibular canine regions within the interforaminal area. Their positions were standardized to be approximately equidistant from the midline, with an inter-implant distance of about 20 ± 2 mm, depending on individual ridge anatomy. A surgical stent was used to guide implant placement, ensuring consistent mesiodistal and buccolingual positioning. Implant parallelism was assessed using paralleling pins to limit angulation discrepancies to within 10°, thereby promoting favorable load distribution for immediately loaded overdentures. Following flap elevation, osteotomy preparation and implant placement were carried out according to standard surgical protocols under copious irrigation. Dental implants (IS-III ACTIVE, Neobiotech, South Korea) measuring 4.0 mm in diameter and 10 mm in length were inserted with a torque of 35–40 Ncm to facilitate immediate loading [31].

**Fig. 2:**
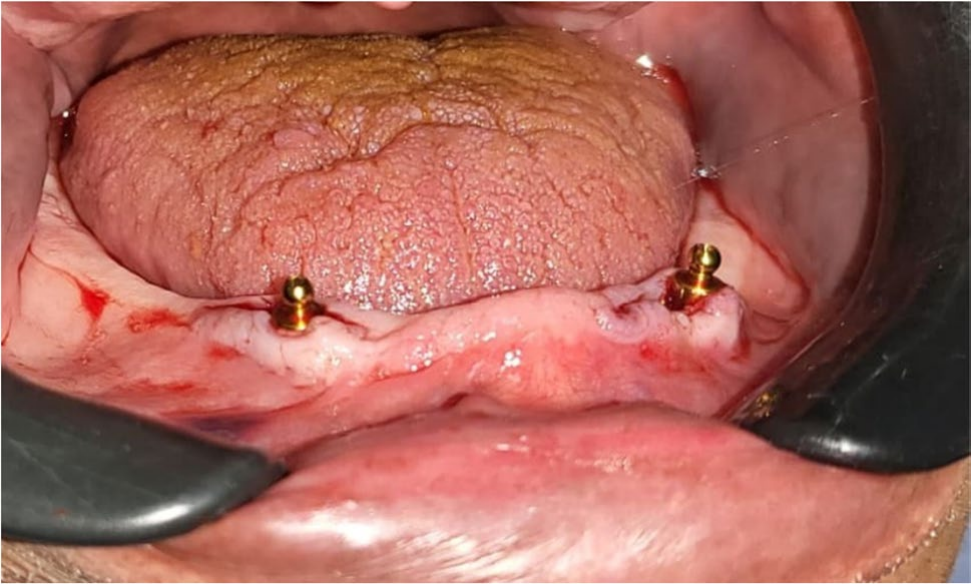
Intraoral view of a participant in the IB group, demonstrating the implant-supported overdenture design with two interforaminal implants with ball attachments

In group IB, ball abutments (IS-III ACTIVE System, Neobiotech Co. Ltd., Seoul, Republic of South Korea) with a diameter of 3.5 mm and a height of 11.5 mm were connected to the implant fixtures. Following abutment placement, the surgical flap was sutured using the interrupted suturing technique (5 − 0 polyglycolic acid sutures, Egy Sorb, Taisier-Med Surgical Sutures and Mesh, Cairo, Egypt) (Fig. 2).

Relief space was then created in the fitting surface of the denture opposite the attachment sites. The sutures were lubricated. The resilient liner (Promedica Soft liner, Promedica Dental Material GmbH, Cuxhaven, Germany) was then mixed and applied to the relevant areas after adhesive application. The patients were asked to close in the centric relation position. After the resilient liner was set, excess material was trimmed, the denture was redelivered to the patients, and follow-up appointments were set for necessary adjustments.

**Fig. 3:**
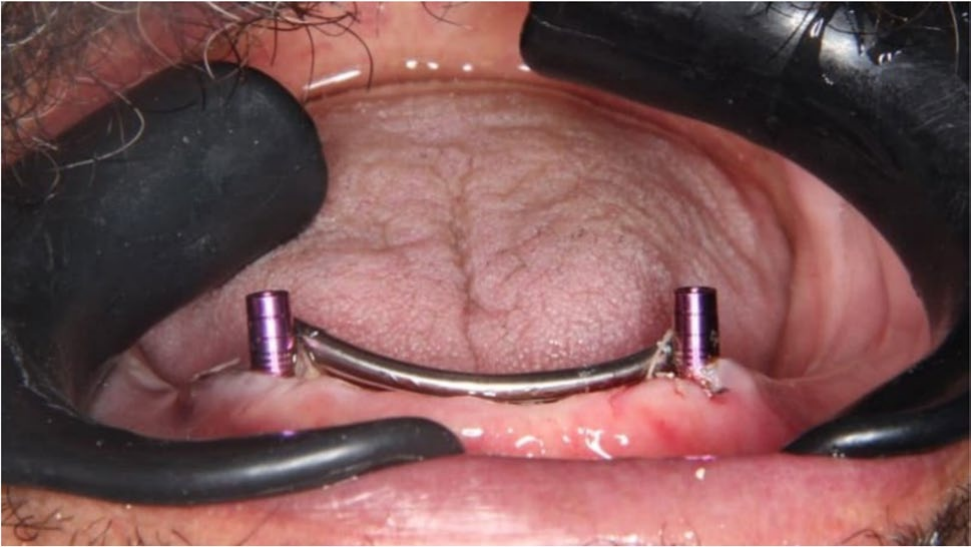
Intraoral view of a participant in the IW group, demonstrating the implant-supported overdenture design with two interforaminal implants with a welded bar

While in the group (IW), the same implant placement protocol was followed. Following implant insertion, titanium standard abutments were attached and tightened according to the manufacturer’s guidelines. A 2 mm readymade, rounded cross-section titanium bar (Guilin Woodpecker Medical Instrument Co., Ltd., China) was then adapted and aligned parallel to the crest of the ridge using a marker and dental pliers. The bar position was adjusted to be 2 mm away from the ridge crest, as verified with a periodontal probe. Before welding, the bar was firmly stabilized in the predetermined position against the abutments using a suitable plier instrument to prevent displacement during the procedure. The abutments were then splinted to the titanium bar using intraoral welding (JD Weld, J Dental Care Srl, Modena, Italy) at 1 pulse and 0.37% power under continuous water coolant irrigation. The welding tip was applied at each bar–abutment junction, oriented perpendicular to the contact interface, while continuous irrigation was maintained to limit heat generation. After completion of the welding procedure, the stability of the splinted framework was clinically assessed. Any irregularities or sharp edges were subsequently smoothed and polished to protect the surrounding soft tissues (Fig. 3).

Similarly, as in the group (IB), sufficient relief space was created, and the resilient liner was applied after blocking the undercut beneath the bar. Standard postoperative medications were prescribed [31].

The T-Scan system (T-Scan III, Tekscan^®^, South Boston, MA, USA) was employed for computerized occlusal adjustment of all participants’ dentures and for recording occlusal parameters. Occlusion time (OT), disocclusion time toward the right side (DR), and disocclusion time toward the left side (DL) were measured on the day of implant placement (T1) and after three months (T2). OT was defined as the interval from the first tooth contact to maximum intercuspation during mandibular closure, whereas DT was defined as the duration from the onset of mandibular excursive movement to complete separation of the posterior teeth.

For each participant, the size of the sensor was determined according to the size of the dental arch, following the manufacturer’s guidelines (sensor thickness approximately 100 μm). For analysis, the participants were instructed to sit in an upright position in the dental chair with a standardized head position, and the sensor was placed parallel to the occlusal plane and centred on the midline of the dental arch. The same sensor was used for each participant during the occlusal adjustment and the T1 and T2 intervals. For calibration, a proper sensitivity range was set. Participants were asked to perform a number of 2 to 4 closures for sensor conditioning, and all the measurements were performed with a single blinded calibrated operator to minimize inter-operator variability [30].

For occlusal corrections, the centre of force concept was utilized. Participants were instructed to bite into maximum intercuspation while wearing the sensor using consistent moderate biting force, maintain that position for 1 to 3 s, then to disocclude and repeat the intercuspation. Premature occlusal contacts were adjusted according to the software’s color-coded maps and force readings until a balanced force distribution was achieved, maintaining approximately 50% ± 2% on each side [30].

**Fig. 4:**
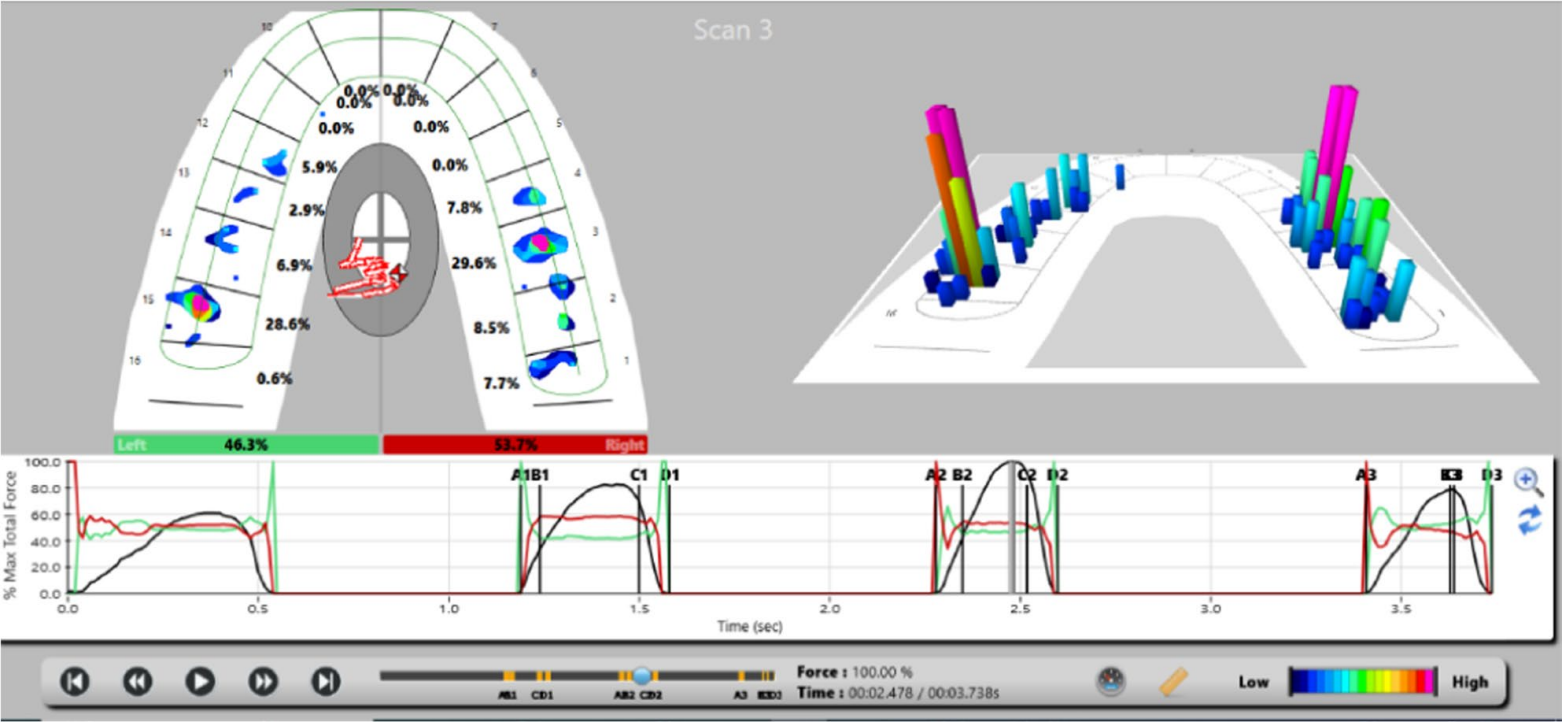
T-Scan analysis demonstrating the occlusion and disocclusion times recorded for a participant in the conventional complete denture group (CD)

For the occlusal parameter (OT), the participants were guided to bite on the sensor with consistent moderate pressure until maximum intercuspation and to keep holding their teeth together for a period of 1 to 3 s, followed by disocclusion (Fig. 4). This step was repeated four times.

For recording the occlusal parameter (DR), participants were instructed to make a right lateral excursion from the position of complete intercuspation, followed by disocclusion. This step was also repeated four times. Similar steps were made to the left side for the occlusal parameter (DL). Mean OT, DR, and DL times were then calculated by taking the averages of the recorded values [27].

Statistical analysis was performed using the Statistical Package for the Social Sciences (version 21.0, SPSS Inc., IBM Corporation, Chicago, USA). The Shapiro-Wilk test revealed a normal distribution of the resultant data. Mixed ANOVA with Greenhouse–Geisser correction was conducted to compare the effect of time (T1 versus T2) and the effect of treatment (between groups), as well as the interaction between time and treatment on the mean values of OT, DR, and DL. The Paired t-test was used for the intra-group comparisons of OT, DR, and DL times in each group between the T1 and T2 intervals. Post hoc Tukey analysis was used for the intergroup comparison between the three groups. *P*- value < 0.05 was the statistical significance level. Bonferroni correction was made for the adjustment of the *P*- value to account for multiple testing across the groups.

## Results

A total of 36 participants, 20 females and 16 males, completed the study, with an age range (53–70) years. The age ranges for Group CD, Group IB, and Group IW were 56–67, 54–70, and 53–68 years, respectively. Group CD included 5 males and 7 females; Group IB involved 4 males and 8 females; and Group IW included 7 males and 5 females. Demographic data of the participants are available in Table 1.

**Table 1:** Assigned group and demographic data for the participants

Patient number	Assigned group	Age(years)	Gender	Period of edentulism (Months)	Socioeconomic status
1	CD	56	Female	6	Upper
2	CD	56	male	10	Lower
3	IB	58	female	11	Lower
4	IW	55	female	8	lower
5	IB	60	Female	5	Upper
6	IB	64	Female	7	intermediate
7	IB	70	Male	12	intermediate
8	CD	67	Female	12	Lower
9	IB	68	Male	8	Intermediate
10	IW	65	Female	9	Lower
11	IW	57	male	9	Intermediate
12	CD	58	Male	8	Upper
13	CD	59	Male	10	Intermediate
14	IB	65	female	7	Upper
15	IB	67	Male	8	Lower
16	IW	66	Male	6	Lower
17	IW	59	male	5	Intermediate
18	IW	68	Female	7	Lower
19	IB	57	female	9	Intermediate
20	CD	64	male	11	Lower
21	IB	69	female	12	Intermediate
22	IB	54	Male	8	Upper
23	IB	66	female	10	Lower
24	CD	63	female	7	Lower
25	CD	61	female	9	Lower
26	IW	53	male	5	Intermediate
27	CD	60	Male	11	Intermediate
28	IW	55	Male	6	Intermediate
29	IB	63	female	12	Intermediate
30	IW	63	male	8	Intermediate
31	IW	60	Female	5	Upper
32	CD	59	female	5	Lower
33	CD	67	female	11	Intermediate
34	IW	64	female	10	Lower
35	CD	64	female	10	Lower
36	IW	54	male	9	Intermediate

CD: group receiving complete denture, IB: group receiving implant overdentures with ball attachments, IW: group receiving implant overdentures with welded

For the OT, DR, and DL parameters, the Mixed ANOVA test showed significant differences among the three groups (CD, IB, and IW), and both intervals (T1 and T2) (*P* < 0.001). In addition, a statistically significant group × time interaction was observed, indicating that changes over time differed significantly between groups Tables 2, 3, and 4).

**Table 2:** Mixed ANOVA results for occlusion time (OT) showing main effects of group, time, and interaction

Source of Variation	Type III Sum of Squares	df	Mean Square	F-value	*P*-value	Partial Eta Squared
Groups	0.343	2	0.171	62.561	< 0.001	0.791
Time	0.025	1	0.025	47.70	< 0.001	0.603
Groups × Time	0.080	2	0.040	13.181	< 0.001	0.444

*df* degree of freedom

**Table 3:** Mixed ANOVA results for disocclusion time right (DR) showing main effects of group, time, and interaction

Source of Variation	Type III Sum of Squares	df	Mean Square	F-value	*P*-value	Partial Eta Squared
Groups	0.404	2	0.202	142.909	< 0.001	0.896
Time	0.041	1	0.041	71.38	< 0.001	0.767
Groups × Time	0.036	2	0.018	8.777	< 0.001	0.347

*df* degree of freedom

**Table 4:** Mixed ANOVA results for disocclusion time Left (DL) showing main effects of group, time, and interaction

Source of Variation	Type III Sum of Squares	df	Mean Square	F-value	*P*-value	Partial Eta Squared
Groups	0.319	2	0.160	78.290	< 0.001	0.826
Time	0.052	1	0.052	40.390	< 0.001	0.550
Groups × Time	0.021	2	0.0105	16.15	< 0.001	0.418

*df* degree of freedom

Post hoc Tukey analysis consistently demonstrated significant differences between Group CD and Group IB (*P* < 0.001, Q = 10.75,14.21,17.27) and between Group CD and Group IW (Q = 14.03,17.02,20.33, *P* < 0.001). However, no significant difference between Group IB and Group IW (Q = 3.29, *P* = 0.06), (Q = 2.8, *P* = 0.13), (Q = 3.05, *P* = 0.09) for the three parameters was detected. For the OT parameter, 95% confidence intervals were (0.48,0.57), (0.31,0.38), and (0.28,0.31) for the groups CD, IB, and IW, respectively. Regarding the DR, the 95% confidence intervals (0.55,0.6), (0.37,0.42), and (0.34,0.39) for the groups CD, IB, and IW, respectively. While in the DL parameter, the 95% confidence intervals (0.56,0.61), (0.42,0.43), and (0.38,0.41) for the groups CD, IB, and IW, respectively. These findings remained similar for the OT, DR, DL parameters after three months, where Group CD continued to differ significantly from both IB (Q = 10.63, 9.13 ,9.54, *P* < 0.001) and IW (Q = 12.95, 12.47,12.74, *P* < 0.001), while IB and IW (Q = 2.32, *P* = 0.24), (Q = 3.34, *P* = 0.64), (Q = 3.19, *P* = 0.07) still showed no significant difference. The 95% confidence intervals in the OT parameter for the groups CD, IB, and IW, respectively, were (0.43,0.51), (0.31,0.35), and (0.28,0.31). While for the DR, the intervals were (0.48,0.54), (0.35,0.41), (0.3,0.36) and (0.5,0.54), (0.39,0.42), and (0.35,0.39) in the DL parameter. The paired t-test revealed a significant change over time only in Group CD, while Groups IB and IW did not show significant changes. Mean and standard deviation values are reported in Tables 5, 6, and 7).

**Table 5:** Mean and standard deviation values for the occlusion time parameter in the groups CD, IB, and IW at T1 and T2 intervals

	Timing of assessment	CD	IB	IW	*P*-value
		X (Sec) ± SD	X (Sec) ± SD	X (Sec) ± SD	
	**T1**	0.52 ± 0.07 ^Aa^	0.35 ± 0.06^Ab^	0.29 ± 0.02 ^Ab^	*P* > 0.001
	**T2**	0.47 ± 0.07^Ba^	0.33 ± 0.03^Ab^	0.28 ± 0.02 ^Ab^	*P* > 0.001
**T-value**		T= -1.91	T= -1.22	T = 0.39	
***P-*** **value**		*P* = 0.04	*P* = 0.12	*P* = 0.35	

Different superscript lowercase letters indicate a significant difference between different groups at the same time interval

Different superscript uppercase letters indicate a significant difference in the same group at different time intervals

*OT* Occlusion time parameter, *X* mean, *SD* Standard deviation, *Sec* Second, *T1* Day of implant placement, *T2* Three months after implant placement

**Table 6:** Mean and standard deviation values for the Disocclusion time to the right-side parameter (DR) in the groups CD, IB, and IW at T1 and T2 intervals

	Timing of assessment	CD	IB	IW	*P*-value
		X (Sec) SD	X (Sec) SD	X (Sec) SD	
	**T1**	0.57 ± 0.04 ^Aa^	0.40 ± 0.04 ^Ab^	0.36 ± 0.03 ^Ab^	*P* > 0.001
	**T2**	0.51 ± 0.05 ^Ba^	0.38 ± 0.05 ^Ab^	0.33 ± 0.04 ^Ab^	*P* > 0.001
**T-value**		T= -3.01	T= -1.08	T= -1.5	
***P*** **-value**		*P* = 0.005	*P* = 0.14	*P* = 0.07	

Different superscript lowercase letters indicate a significant difference between different groups at the same time interval

Different superscript uppercase letters indicate a significant difference in the same group at different time intervals

*DR* Disocclusion time to the right-side parameter, *X* mean, *SD* Standard deviation, *Sec* Second, *T1* Day of implant placement, *T2* Three months after implant placement

**Table 7:** Mean and standard deviation values for the Disocclusion time to the left side (DL) parameter in the groups CD, IB, and IW at T1 and T2 intervals

	Timing of assessment	CD	IW	IB	*P*-value
		X (Sec) ± SD	X (Sec) ± SD	X (Sec) ± SD	
	**T1**	0.58 ± 0.04 ^Aa^	0.40 ± 0.02 ^Ab^	0.42 ± 0.01 ^Ab^	*P* > 0.001
	**T2**	0.52 ± 0.04 ^**B**a^	0.37 ± 0.04 ^Ab^	0.41 ± 0.03 ^Ab^	*P* > 0.001
**T-value**		T= -4.1	T= -1.5	T= -1.5	
***P*** **-value**		*P* = 0.0008	*P* = 0.07	*P* = 0.07	

Different superscript lowercase letters indicate a significant difference between different groups at the same time interval

Different superscript uppercase letters indicate a significant difference in the same group at different time intervals

*DL* Disocclusion time to the left side parameter, *X* mean, *SD* Standard deviation, *Sec* Second, *T1* Day of implant placement, *T2* Three months after implant placement

## Discussion

This study aims to evaluate OT and DT in immediately loaded implant overdentures retained by two interforaminal implants, comparing IW and IB designs, with CD included as a control group. The objective was to examine the effect of attachment design on functional occlusal forces and occlusal performance under immediate loading [32–34]. The null hypothesis of this study was partially rejected, as significant differences were found between both IW and IB groups when compared to the CD group regarding the OT and DT. However, no significant difference was detected between IW and IB.

Smokers were excluded due to the negative impact of smoking on tissue healing and implant osseointegration under immediate-loading protocols [35, 36]. Furthermore, patients who were completely edentulous in both the maxilla and mandible were selected to reduce implant loading by using a maxillary complete denture and adjusting the occlusal scheme [37].

In this study, implant placement was performed using an open-flap approach after CBCT assessment with a radiographic stent. Flap elevation enabled direct visualization of the ridge for implant positioning in the canine area. Although guided flapless techniques may improve positional precision, clinical outcomes have been reported to be comparable to the open-flap method. However, a clear surgical acrylic stent was used to control the implant positioning and interimplant distance. Crestal bone levels were checked to be the same on both sides, indicating similar coronal-apical positioning of the implant [38].

In the present study, retention to attachments was achieved using a soft relining material without incorporating the definitive matrices. This approach was applied during the immediate loading phase to allow functional adaptation of the overdenture to the attachments. The resilient lining material may also help decrease occlusal forces transmitted to the implants during early loading [39–41].

OT and DT were recorded using the T-Scan system under standardized conditions to ensure measurement reliability and precision [19, 34, 37]. Additionally, all occlusal adjustments and measurements were performed with the patient seated upright, as the sagittal plane alignment of the head and neck in this position can significantly influence initial occlusal contacts and, consequently, the accuracy of the results [42, 43].

The findings of this study revealed significantly shorter OT and DT in both IW and IB groups than in the CD group, indicating greater occlusal efficiency and stability due to the rigid implant foundation, compared with tissue-supported complete dentures, which rely on resilient, compressible supporting tissues that require time for settling and adaptation. Moreover, the greater resilience of the mucosa under complete dentures than under implant-supported overdentures may have contributed to shorter OT and DT in both IW and IB groups than in the CD group [24, 44]. These results may also be explained by a previous randomized clinical trial, which showed that implant configuration and attachment design influence load distribution, occlusal stability, and peri-implant bone preservation, underscoring the importance of careful planning of overdenture design [45].

Despite the IW group in this study showing slightly lower OT and DT values than IB, the difference was not significant. This may be explained by the fact that, in two-implant overdentures, force distribution is largely controlled by the supporting bone and denture base, which can limit the additional effect of splinting, resulting in similar functional behaviour in both designs [31, 46]. Additionally, the relatively small diameter and dimensions of the titanium bar, along with its rounded cross-section and flexibility, may have contributed to the finding that splinted attachments did not perform better than unsplinted ones. Clinically, this suggests that splinting of two implants in an overdenture may not provide a clear functional advantage in such cases, and attachment selection can be based on practical considerations such as simplicity, maintenance, and cost.

The study results aligned with prior research, which demonstrated that implant overdentures achieve significantly shorter OT and DT values compared to conventional complete dentures [24]. In the present study, the OT and DT values were OT: 0.29–0.52 s, DR: 0.33–0.57 s, and DL: 0.37–0.58 s, consistent with values reported in earlier studies [24, 44, 47].

During follow-up, both OT and DT significantly decreased in the CD group, likely due to denture base settling, tissue adaptation, and improved stability-enhancing occlusal contacts [43, 27]. One more study also showed a significant reduction in the OT, DR, and DL values after 3 months of complete denture insertion; a finding that was justified in the light of adaptation and improved stability [44]. Whenever the stability of dentures is improved, the occlusal parameters are reduced, as concluded by Kabbua et al. [27]. In contrast, the IW and IB groups showed non-significant changes in mean values, which may be explained by their initially Shorter OT and DT resulting from the rigid implant foundation, leading to minimal variation over time [47].

This study has several limitations. The sample size was calculated based on maximum occlusal force (MOF) rather than OT and DT because data on these parameters in implant overdentures were limited. However, these outcomes are conceptually related, as MOF reflects the total force during occlusion and is inherently linked to occlusal efficiency and stability, while OT serves as an indicator of occlusal performance and masticatory function. Moreover, the current study lacks comprehensive blinding as blinding of participants and surgical operators was not feasible, as only the T-Scan assessor was blinded. Furthermore, implant stability was evaluated using insertion torque values without measuring the implant stability quotient (ISQ), as the Osstell^®^ measuring device was not feasible during the study. Nevertheless, a systematic review recommended implant lengths of 8–11 mm and insertion torque values of 30–45 Ncm for immediate loading, criteria that were fulfilled in the current study, and reported that most included studies relied on insertion torque rather than ISQ [48]. Additionally, the three-month follow-up period was relatively short for assessing long-term functional adaptation and occlusal stability. Patient-reported outcomes as well as full clinical simulation of function were not evaluated either. Although intraoral welding showed promising results, it is technique-sensitive and may increase clinical complexity. Therefore, further well-designed clinical trials with longer follow-up periods are recommended.

## Conclusion

Within three months of follow-up, immediately loaded mandibular overdentures retained by two implants demonstrated similar occlusion and disocclusion times for splinted intraoral welded bar and non-splinted ball attachments, while both attachment types resulted in shorter times compared to a conventional complete denture under the tested conditions.

## Supplementary Information

Supplementary Material 1

## Data Availability

The data generated and analysed in this research can be accessed through the corresponding author upon reasonable request.

## Abbreviations

OT: Occlusion time
DT: Disocclusion time
CD: Conventional Complete Dentures
IB: Implant retained overdentures retained by ball attachments
IW: Implant retained overdentures retained by intraoral welded bar
CBCT: Cone Beam Computed Tomography
DR: Disocclusion time towards the right side
DL: Disocclusion time towards the left side
T1: Day of implant placement
T2: Three months after implant placement
